# Association between preoperative geriatric nutritional risk index and early postoperative cognitive decline after laparoscopic cholecystectomy

**DOI:** 10.3389/fnut.2026.1905999

**Published:** 2026-10-20

**Authors:** Yanlong Fu, Qiang Wei, Wenxin Shi

**Affiliations:** 1 Department of Hepatobiliary Surgery, The Bethune International Peace Hospital, Shijiazhuang, Hebei, China; 2 Graduate School of North China University of Technology, North China University of Science and Technology, Hebei, China; 3 Department of Obstetrics and Gynecology, The Second Hospital of Hebei Medical University, Shijiazhuang, Hebei, China

**Keywords:** early postoperative cognitive decline (ePOCD), Geriatric Nutritional Risk Index (GNRI), IL-6, laparoscopic cholecystectomy, Perioperative outcomes

## Abstract

**Background:**

Early postoperative cognitive decline (ePOCD) is a frequent complication in elderly surgical patients. The association between preoperative nutritional status and early postoperative cognitive decline remains unclear. This study investigated the relationship between preoperative Geriatric Nutritional Risk Index (GNRI) and early postoperative cognitive decline in elderly patients undergoing laparoscopic cholecystectomy.

**Objective:**

To investigate the association between preoperative GNRI and ePOCD in older patients undergoing laparoscopic cholecystectomy, and to explore the discriminatory ability of GNRI for ePOCD and its association with postoperative inflammatory markers.

**Methods:**

This study was a retrospective secondary analysis of a prospectively collected observational cohort. A total of 361 elderly patients who underwent laparoscopic cholecystectomy were included. Preoperative nutritional status was assessed using the GNRI. Cognitive function was evaluated before surgery and on postoperative days 1 and 2 using standardized cognitive assessments. Early postoperative cognitive decline was defined as a decrease of ≥3 points from the preoperative baseline in either the MMSE or MoCA score. Patients were categorized according to GNRI nutritional risk groups, and associations between preoperative GNRI and ePOCD were analyzed using logistic regression models.

**Results:**

Among the 361 patients, 24 (6.6%) experienced early postoperative cognitive decline during the early postoperative period. The incidence of cognitive decline was higher on postoperative day 1 than on postoperative day 2. Patients with early postoperative cognitive decline had lower preoperative GNRI values compared with those without cognitive decline. After adjustment for potential confounding factors, lower GNRI remained associated with an increased risk of early postoperative cognitive decline. Older age was also independently associated with increased risk. Exploratory analyses demonstrated differences in perioperative inflammatory and neuronal injury-related biomarkers among GNRI groups; however, these findings require further validation.

**Conclusion:**

Lower preoperative GNRI was associated with an increased risk of early postoperative cognitive decline in elderly patients undergoing laparoscopic cholecystectomy. GNRI may serve as a simple indicator for identifying elderly patients with increased vulnerability to early postoperative cognitive changes. Further prospective multicenter studies and interventional trials are required to determine whether improving preoperative nutritional status can modify postoperative cognitive outcomes.

## Introduction

Laparoscopic cholecystectomy is a common minimally invasive surgical procedure used for the treatment of gallbladder diseases. It offers advantages including reduced surgical trauma, faster recovery, and a low incidence of complications, and is increasingly performed in elderly patients. However, older adults often exhibit decreased physiological reserve, impaired nutritional status, and reduced cognitive resilience, which may increase their susceptibility to postoperative complications. Early postoperative cognitive decline(ePOCD)is a frequently observed complications after surgery in older patients, and is characterized by deterioration in cognitive performance involving memory, attention, executive function, and orientation during the early postoperative period (1–6). Although some patients may recover over time, early postoperative cognitive changes may represent an important clinical concern because they are associated with delayed recovery and may contribute to subsequent cognitive vulnerability.

The mechanisms underlying postoperative cognitive changes remain incompletely understood. Previous studies have suggested that multiple factors, including advanced age, anesthesia-related factors, advanced surgical stress, inflammation, and nutritional status (7–14). Among these factors, nutritional status, may contribute to postoperative cognitive alterations. Elderly patients are particularly vulnerable to malnutrition or nutritional risk due to reduced appetite, impaired digestion and absorption, and underlying comorbidities. Nutritional impairment may compromise immune function, reduce physiological stress tolerance, and increase susceptibility to adverse postoper-ative outcomes.

The GNRI is a nutritional assessment tool specifically developed for older adults. Its incorporates serum albumin concentration and body weight and has the advantages of simplicity, accessibility, and reproducibility, making it widely applicable for evaluating nutritional status and prognosis in elderly populations.

Previous studies have demonstrated that GNRI is associated with postoperative complications and clinical outcomes among elderly surgical patients (15–17). However, imited evidence is available regarding the relationship between preoperative GNRI and ePOCD in elderly patients undergoing laparoscopic cholecystectomy. In addition, although our previous work explored perioperative factors potentially associated with postoperative cognitive outcomes, including anesthetic-related variables (18), the relationship between preoperative nutritional status and early postoperative cognitive changes remains insufficiently characterized.

Therefore, this study aimed to investigate the association between preoperative GNRI and ePOCD in older patients undergoing laparoscopic cholecystectomy and to explore the discriminatory ability of GNRI for ePOCD and its association with postoperative inflammatory markers.

## Methods

### Study design and participants

This was a retrospective secondary analysis of a prospectively collected observational cohort conducted at the Department of hepatobiliary surgery, Bethune International Peace Hospital, between July 2023 and July 2024. The cohort was initially established to investigate POCD after laparoscopic cholecystectomy. The present study included all 361 patients from this cohort, including 165 patients who had been included in our previous publication (18). Because the present study was a secondary analysis of an established cohort, no independent sample size calculation was performed. The sample size was determined by the number of eligible participants available from the original prospective cohort. Given the limited number of early postoperative cognitive decline events, multivariable models were restricted to clinically relevant variables to minimize overfitting. A total of 361 elderly patients aged 60–80 years under general anesthesia with American Society of Anesthesiologists (ASA) physical status I–II were enrolled. Patients were excluded if they had preoperative cognitive impairment, major psychiatric or neurological disorders, severe visual, auditory, or language dysfunction, alcohol or substance abuse, pre-existing systemic inflammatory disease, emergency surgery, severe hepatic or renal dysfunction, or other conditions that could interfere with cognitive or inflammatory assessments. The study protocol was approved by the Ethics Committee of Bethune International Peace Hospital (Ethics Approval No.: 2021-KY-910), and written informed consent was obtained from all participants or their legal representatives prior to enrollment.

### Data collection

Baseline demographic, clinical, and surgical variables were collected for all patients. Demographic data included age, gender, education years, and body mass index (BMI). Clinical data comprised ASA physical classification, preoperative Mini-Mental State Examination (MMSE) score, and Montreal Cognitive Assessment (MoCA) score. Surgical and anesthetic data included operation duration, anesthesia duration, estimated intraoperative blood loss, anesthetic agents, and intraoperative bispectral index monitoring. Postoperative pain score on day 1, postoperative complications, and length of hospital stay were also documented. All data were extracted from a prospectively maintained database, double-entered by two independent investigators, cross-checked for consistency, and verified by a third reviewer in case of discrepancies.

#### Assessment of early postoperative cognitive decline (ePOCD)

Cognitive function was assessed using the Mini-Mental State Examination (MMSE) and the Montreal Cognitive Assessment (MoCA) before surgery and on postoperative days 1 and 2. All cognitive evaluations were performed by trained clinicians who were blinded to the patients' nutritional status and followed a standardized assessment protocol. Because cognitive assessments were limited to the early postoperative period, the primary outcome of the present study was early postoperative cognitive decline rather than persistent postoperative neurocognitive disorder.

According to the predefined protocol of the original prospective cohort and the Chinese Guidelines for the Diagnosis and Treatment of Dementia and Cognitive Disorders, early postoperative cognitive decline was defined as a decrease of 3 or more points in either the MMSE or MoCA compared with the preoperative baseline score. Cognitive decline was evaluated separately on postoperative day 1 and postoperative day 2, and patients meeting the criterion at either postoperative assessment were considered to have experienced early postoperative cognitive decline during the early postoperative period.

### Geriatric Nutritional Risk Index (GNRI)

GNRI was calculated as: 1.489 × serum albumin (g/L) + 41.7 × (actual body weight / ideal body weight). Ideal body weight was calculated using the Lorentz formula. Patients were classified into three nutritional risk groups: low risk (GNRI ≥ 98), moderate risk (92 ≤ GNRI < 98), and high risk (GNRI < 92). For subgroup analysis, GNRI was dichotomized as high risk (GNRI < 92) vs. low risk (GNRI ≥ 98). These calculations were based on data collected at baseline during the original study.

### Laboratory measurements

Peripheral venous blood specimens were harvested at baseline (1 day preoperatively) and on postoperative day POD 1 and POD 2. Serum concentrations of interleukin-6 (IL-6), tumor necrosis factor-α (TNF-α), interleukin-1β (IL-1β), C-reactive protein (CRP), D-lactic acid, lipopolysaccharide (LPS), zonulin, S100β, and neuron-specific enolase (NSE) were quantified following routine standardized laboratory protocols. Changes in IL-6 concentration (ΔIL-6) were calculated as the difference between POD 2 and preoperative baseline values.

### Enzyme-linked immunosorbent assay (ELISA)

All measurements were performed using commercially available ELISA kits (Sigma-Aldrich Trading Co., Ltd., Shanghai, China for IL-6, TNF-α, S100β, CRP, and IL-1β; Thermo Fisher Scientific (China) Co., Ltd., Shanghai, China for Zonulin and NSE; Shanghai Univ-bio Co., Ltd., Shanghai, China for D-lactic acid; Wuhan Fine Biotech Co., Ltd., Wuhan, China for LPS) strictly following the manufacturer's instructions. In brief, 100 μL of diluted serum samples, standards, and controls were added to pre-coated 96-well plates and incubated for 2 h at 37 °C. After washing, 100 μL of biotinylated detection antibody was added and incubated for 1 h, followed by streptavidin-horseradish peroxidase (HRP) incubation for 30 min. After a final wash, 100 μL of tetramethylbenzidine (TMB) substrate was added and incubated for 15 min in the dark. The reaction was stopped with 50 μL of stop solution, and the optical density was read at 450 nm using a microplate reader. Sample concentrations were calculated from standard curves generated by four-parameter logistic regression.

### Statistical analysis

Statistical analyses were performed using SPSS version 26.0 and R software version 4.3.0. Continuous variables are presented as mean ± standard deviation (SD) or median (interquartile range [IQR]), as appropriate, and categorical variables as n (%). Comparisons among GNRI groups were performed using one-way analysis of variance (ANOVA), the Kruskal–Wallis test, or the chi-square test/Fisher's exact test, as appropriate. All statistical tests were two-sided, and a *P* value < 0.05 was considered statistically significant.

Longitudinal MMSE scores were analyzed using a linear mixed-effects model, with GNRI group, time (preoperative, POD1, and POD2), and the GNRI group × time interaction included as fixed effects and participant included as a random effect. Postoperative IL-6 and S100β levels were analyzed using baseline-adjusted linear regression models, with the corresponding preoperative biomarker concentration and prespecified clinical covariates included as covariates.

Given the limited number of ePOCD events, Firth penalized logistic regression was used for univariable and multivariable analyses to reduce small-sample bias and potential separation. Parsimonious multivariable models were constructed using clinically relevant covariates to minimize overfitting. GNRI was evaluated separately as a continuous variable and as a categorical variable. Odds ratios (ORs), 95% confidence intervals (CIs), and corresponding *P* values are reported.

The primary multivariable model was internally validated using 1,000 bootstrap resamples. Model performance was assessed using the apparent and optimism-corrected area under the receiver operating characteristic curve (AUC) and the optimism-corrected calibration slope. Spearman rank correlation analysis was used to assess the association between preoperative GNRI and POD2 IL-6 levels, with Spearman's ρ, 95% CI, and corresponding *P* value reported.

Exploratory subgroup analyses were performed using Firth penalized logistic regression, with interaction terms used to assess potential effect modification. Given the limited number of ePOCD events and multiple subgroup comparisons, these analyses were considered exploratory and hypothesis-generating.

## Results

Between July 2023 and July 2024, a total of 366 patients were assessed for eligibility. Of these, five patients were excluded for the following reasons: medication-use conflicts (*n* = 2), limited cognitive ability (*n* = 1), a specific comorbidity meeting the exclusion criteria (*n* = 1), and postoperative admission to the intensive care unit (ICU) with subsequent loss to follow-up (*n* = 1). Consequently, 361 patients were included in the final analysis. Complete data for all major variables were available for all included patients. The participant selection process is shown in Figure 1.

**Figure 1:**
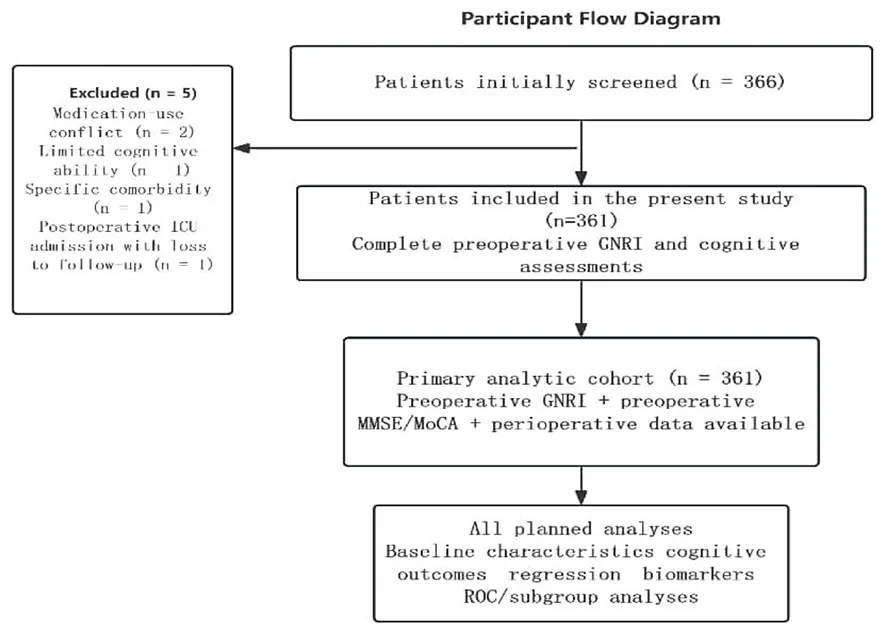
Flow diagram.

### Baseline characteristics

Among the 361 included patients, 152 were classified as low GNRI risk (GNRI ≥ 98), 128 as moderate risk (92 ≤ GNRI < 98), and 81 as high risk (GNRI < 92). Most demographic and perioperative characteristics were comparable among the three groups. Preoperative MMSE scores differed modestly across GNRI categories (*P* = 0.025), whereas preoperative MoCA scores were comparable (*P* = 0.157). Serum albumin levels decreased progressively with increasing GNRI risk (*P* < 0.001) (Table 1).

**Table 1:** Comparison of baseline data among different GNRI groups.

Variable	Low risk GNRI ≥98 (*n* = 152)	Moderate risk 92 ≤ GNRI < 98 (*n* = 128)	High risk GNRI < 92 (*n* = 81)	*P* value
Age, years	70.7 ± 6.9	70.3 ± 6.6	71.7 ± 7.2	0.355
Male sex, n (%)	75 (49.3%)	63 (49.2%)	42 (51.9%)	0.943
Years of education	7.9 ± 3.3	8.1 ± 3.2	7.7 ± 3.5	0.661
BMI, kg/m^2^	26.2 (23.7–29.3)	25.8 (23.5–28.9)	25.6 (23.3–28.7)	0.632
Preoperative MMSE score	28.5 ± 1.4	28.4 ± 1.5	27.6 ± 1.7	0.025
Preoperative MoCA score	27 (25–28)	27 (24–28)	26 (24–28)	0.157
ASA I, n (%)	82 (53.9%)	68 (53.1%)	39 (48.1%)	0.671
ASA II, n (%)	70 (46.1%)	60 (46.9%)	42 (51.9%)	
Operation duration, min	88.4 ± 20.3	90.9 ± 21.5	91.7 ± 19.5	0.542
Anesthesia duration, min	135 (126–154)	139 (126–157)	143 (127–159)	0.444
Intraoperative blood loss, mL	10 (5–15)	10 (5–20)	15 (10–20)	0.086
Postoperative hospital stay, days	3.8 ± 1.2	4.0 ± 1.3	4.2 ± 1.1	0.056
Serum albumin, g/L	40.1 ± 2.8	37.0 ± 3.0	33.8 ± 3.2	< 0.001
GNRI value	101.6 ± 2.4	95.1 ± 1.6	89.2 ± 2.1	—

Data are presented as mean ± standard deviation, median (interquartile range), or n (%), as appropriate. Continuous variables were compared using one-way analysis of variance (ANOVA) or the Kruskal–Wallis test, as appropriate. Categorical variables were compared using the chi-square test or Fisher's exact test, as appropriate. GNRI, Geriatric Nutritional Risk Index; BMI, body mass index; MMSE, Mini-Mental State Examination; MoCA, Montreal Cognitive Assessment; ASA, American Society of Anesthesiologists.

### Early postoperative cognitive decline and longitudinal MMSE scores

Overall, 24 of 361 patients (6.6%) experienced ePOCD during the first 2 postoperative days. The incidence increased across GNRI risk categories, from 1.3% (2/152) in the low-risk group to 6.3% (8/128) in the moderate-risk group and 17.3% (14/81) in the high-risk group (*P* < 0.001). Sixteen patients (4.4%) met the ePOCD criterion on POD1 and nine (2.5%) on POD2; one patient met the criterion at both time points and was counted only once in the overall incidence (Table 2).

**Table 2:** Incidence of early postoperative cognitive decline and longitudinal MMSE scores according to GNRI category.

Early postoperative cognitive decline					
Outcome	Total n = 361	Low risk n = 152	Moderate risk n = 128	High risk n = 81	*P*
ePOCD, POD1	16 (4.4%)	1 (0.7%)	7 (5.5%)	8 (9.9%)	0.004
ePOCD, POD2	9 (2.5%)	1 (0.7%)	1 (0.8%)	7 (8.6%)	< 0.001
Any ePOCD during POD1–2	24 (6.6%)	2 (1.3%)	8 (6.3%)	14 (17.3%)	< 0.001
P values were calculated using Fisher's exact test.					
MMSE scores					
Outcome	Total	Low risk	Moderate risk	High risk	
Preoperative	28.4 ± 1.5	28.5 ± 1.4	28.4 ± 1.5	27.6 ± 1.7	
POD1	26.5 ± 1.6	27.6 ± 1.2	26.3 ± 1.5	24.5 ± 1.9	
POD2	27.7 ± 1.7	28.7 ± 0.9	27.6 ± 1.3	25.8 ± 1.4	
Δ1	−1.9 ± 1.4	−1.5 ± 0.6	−2.1 ± 0.7	−2.6 ± 0.7	
Δ2	−0.8 ± 1.5	−0.4 ± 0.1	−0.8 ± 0.5	−1.4 ± 0.5	
Linear mixed-effects model: GNRI group effect, *P* = 0.012; time effect, *P* = 0.029; GNRI group × time interaction, *P* = 0.610.					

Data are presented as n (%) or mean ± SD. ePOCD was defined as a decrease of ≥3 points from the preoperative baseline in either MMSE or MoCA. One patient met the ePOCD criterion on both POD1 and POD2; therefore, the number of unique patients with ePOCD was lower than the sum of the POD1 and POD2 event counts. Longitudinal MMSE scores were analyzed using a linear mixed-effects model including GNRI group, time, and the GNRI group × time interaction as fixed effects and patient as a random effect.

MMSE scores were lower in patients with higher GNRI risk and showed postoperative changes over time. In the linear mixed-effects model, significant main effects were observed for GNRI group (*P* = 0.012) and time (*P* = 0.029), whereas the GNRI group × time interaction was not statistically significant (*P* = 0.610). These findings indicate that MMSE scores differed among GNRI groups and changed over time, but there was no evidence that the longitudinal pattern of MMSE change differed among the groups.

In univariable Firth penalized logistic regression analyses, higher preoperative GNRI was associated with lower odds of ePOCD when GNRI was analyzed as a continuous variable. When GNRI was analyzed categorically, lower nutritional-risk categories were also associated with lower odds of ePOCD compared with the high-risk group. Older age, ASA class II, longer operation duration, and higher POD1 pain scores were associated with higher odds of ePOCD, whereas higher preoperative MoCA scores were associated with lower odds of ePOCD (Table 3).

**Table 3:** Univariable Firth penalized logistic regression analysis of factors associated with ePOCD.

Variable	OR (95% CI)	*P* value
GNRI, per 1-point increase	0.88 (0.84–0.97)	0.009
GNRI category		
High risk (GNRI < 92)	Reference	—
Moderate risk (92 ≤ GNRI < 98)	0.74 (0.57–0.96)	0.021
Low risk (GNRI ≥ 98)	0.71 (0.53–0.97)	0.030
Age, per 5-year increase	1.28 (1.12–1.46)	*P* < 0.001
ASA class II vs I	1.35 (1.01–1.80)	0.043
Preoperative MoCA, per 1-point increase	0.85 (0.79–0.92)	< 0.001
Operation duration, per 15-min increase	1.46 (1.23–1.69)	*P* < 0.001
POD1 pain score, per 1-point increase	1.29(1.04–1.60)	0.017

Data are presented as odds ratios (ORs) with 95% confidence intervals (CIs). GNRI was evaluated separately as a continuous variable and as a categorical variable. For categorical GNRI, the high-risk group (GNRI < 92) was used as the reference category. OR, odds ratio; CI, confidence interval; GNRI, Geriatric Nutritional Risk Index; ASA, American Society of Anesthesiologists; MoCA, Montreal Cognitive Assessment; NRS, Numerical Rating Scale; POD1, postoperative day 1; ePOCD, early postoperative cognitive decline.

#### Multivariable analysis

Given the limited number of outcome events, parsimonious multivariable models using Firth penalized logistic regression were constructed. When GNRI was modeled as a continuous variable, higher GNRI remained independently associated with lower odds of ePOCD after adjustment for age, ASA classification, and preoperative MoCA score (adjusted OR per 1-point increase, 0.91; 95% CI, 0.86–0.96; *P* = 0.001). Older age and lower preoperative MoCA scores were also independently associated with ePOCD, whereas ASA classification was not statistically significant after adjustment. When GNRI was analyzed categorically in a separate model, both the moderate-risk (adjusted OR, 0.39; 95% CI, 0.15–0.98; *P* = 0.046) and low-risk groups (adjusted OR, 0.10; 95% CI, 0.02–0.48; *P* = 0.004) had lower odds of ePOCD than the high-risk group (Table 4).

**Table 4:** Multivariable Firth penalized logistic regression analysis of factors associated with ePOCD.

Model A. GNRI modeled as a continuous variable			
Variable	Adjusted OR	95% CI	*P* value
GNRI, per 1-point increase	0.91	0.86–0.96	0.001
Age, per 5-year increase	1.18	1.02–1.37	0.027
ASA class II vs I	1.24	0.80–1.92	0.334
Preoperative MoCA, per 1-point increase	0.88	0.80–0.97	0.010
Model B. GNRI modeled as a categorical variable			
Variable	Adjusted OR	95% CI	*P* value
GNRI category			
High risk (GNRI < 92)	Reference	—	—
Moderate risk (92 ≤ GNRI < 98)	0.39	0.15–0.98	0.046
Low risk (GNRI ≥ 98)	0.10	0.02–0.48	0.004
Age, per 5-year increase	1.17	1.01–1.36	0.039
ASA class II vs I	1.22	0.78–1.90	0.382
Preoperative MoCA, per 1-point increase	0.89	0.81–0.98	0.019

Adjusted odds ratios (ORs) and 95% confidence intervals (CIs) were estimated using Firth penalized logistic regression because of the limited number of ePOCD events. GNRI was evaluated in two separate multivariable models: as a continuous variable in Model A and as a categorical variable in Model B. The high-risk GNRI group (GNRI < 92) was used as the reference category in Model B. GNRI, Geriatric Nutritional Risk Index; ASA, American Society of Anesthesiologists; MoCA, Montreal Cognitive Assessment; ePOCD, early postoperative cognitive decline.

Internal validation using 1,000 bootstrap resamples indicated some optimism in model performance. The apparent AUC was 0.81, with an estimated optimism of 0.07, yielding an optimism-corrected AUC of 0.74. The optimism-corrected calibration slope was 0.89, suggesting some degree of overfitting.

### Perioperative biomarker profiles

Preoperative levels of several inflammatory, intestinal-barrier, and neuronal-injury biomarkers differed across GNRI groups, with generally higher concentrations observed in patients with higher GNRI risk (Table 5). IL-6 levels increased on POD1 and subsequently decreased on POD2, although they remained higher than preoperative levels.

**Table 5:** Perioperative biomarker profiles and baseline-adjusted postoperative biomarker analyses according to GNRI category.

Panel A. Descriptive biomarker levels according to GNRI category					
Biomarker	Total (*n* = 361)	Low risk (*n* = 152)	Moderate risk (*n* = 128)	High risk (*n* = 81)	*P* value
Inflammatory markers					
Preoperative IL-6, pg/mL	3.4 (2.2–5.0)	2.5 (1.8–3.5)	3.2 (2.3–4.5)	4.6 (3.2–6.8)	0.021
POD1 IL-6, pg/mL	8.5 (5.4–14.2)	6.2 (4.3–9.4)	8.4 (5.7–13.6)	15.0 (9.6–24.0)	0.011
POD2 IL-6, pg/mL	5.9 (3.6–9.5)	4.2 (2.8–6.3)	5.7 (3.7–8.9)	10.4 (6.4–18.1)	0.019
ΔIL-6, POD2 – baseline, pg/mL	2.2 (0.6–4.2)	1.5 (0.5–2.8)	2.1 (0.7–3.8)	4.5 (2.1–8.4)	< 0.001
Preoperative TNF-α, pg/mL	2.8 (1.8–4.2)	2.3 (1.7–3.3)	2.8 (2.0–3.9)	3.6 (2.5–5.5)	0.015
Preoperative IL-1β, pg/mL	1.2 (0.8–1.6)	1.0 (0.7–1.3)	1.2 (0.9–1.5)	1.6 (1.1–2.2)	0.022
Preoperative CRP, mg/L	2.5 (1.4–5.6)	1.8 (0.8–3.1)	2.5 (1.7–5.3)	4.2 (2.2–8.5)	0.028
Intestinal-barrier markers					
Preoperative D-lactic acid, μmol/L	8.2 (6.2–11.8)	7.0 (5.4–9.2)	8.6 (6.6–10.4)	11.1 (8.7–15.8)	0.039
Preoperative LPS, EU/mL	0.33 (0.22–0.55)	0.25 (0.16–0.35)	0.31 (0.23–0.46)	0.48 (0.36–0.74)	0.037
Preoperative zonulin, ng/mL	45.0 (32.2–62.0)	38.4 (28.1–52.0)	44.2 (32.7–58.5)	57.8 (42.0–78.2)	0.013
Neuronal-injury markers					
Preoperative S100β, pg/mL	84 (62–115)	72 (55–95)	84 (62–110)	108 (82–145)	0.025
POD1 S100β, pg/mL	145 (108–195)	118 (92–152)	142 (110–185)	195 (148–262)	0.029
Preoperative NSE, ng/mL	9.5 (7.2–12.5)	8.5 (6.5–10.8)	9.4 (7.2–12.1)	11.5 (8.8–15.0)	0.037
Panel B. Baseline-adjusted postoperative biomarker analyses					
Outcome	Comparison		Adjusted β (95% CI)		*P* value
POD1 IL-6	Moderate vs low GNRI risk		+1.45 (0.39 to 2.48)		0.009
POD1 IL-6	High vs low GNRI risk		+5.34 (3.75 to 6.89)		*P* < 0.001
POD2 IL-6	Moderate vs low GNRI risk		+1.20 (0.21 to 2.04)		0.018
POD2 IL-6	High vs low GNRI risk		+4.28 (2.87 to 5.83)		*P* < 0.001
POD1 S100β	Moderate vs low GNRI risk		+9.6 (−3.4 to 22.6)		0.148
POD1 S100β	High vs low GNRI risk		+18.67(1.8 to 35.4)		0.031

After adjustment for baseline IL-6 concentration and prespecified clinical covariates, the high-risk GNRI group had higher postoperative IL-6 levels than the low-risk group on both POD1 and POD2. Baseline-adjusted analyses of POD1 S100β also showed a higher level in the high-risk group than in the low-risk group, whereas the moderate-risk vs. low-risk comparison was not statistically significant (Table 5).

### Exploratory subgroup analyses

Exploratory subgroup analyses were performed according to age, sex, ASA classification, operation duration, and preoperative MoCA score (Table 6). Given the limited number of ePOCD events and the presence of zero-event cells in several strata, subgroup-specific adjusted ORs were estimated using Firth penalized logistic regression. A nominal interaction was observed for age (*P* for interaction = 0.042), whereas no statistically significant interactions were observed for the other examined subgroups. Given the limited number of ePOCD events and multiple subgroup comparisons, these findings should be considered exploratory and hypothesis-generating.

**Table 6:** Exploratory subgroup analysis of the association between GNRI category and early postoperative cognitive decline.

Subgroup	Low-risk GNRI ePOCD, n/N (%)	High-risk GNRI ePOCD, n/N (%)	Adjusted OR (95% CI)	*P* for interaction
Age				0.042
< 75 years	2/112 (1.8%)	7/45 (15.6%)	0.18 (0.04–0.81)	
≥75 years	0/40 (0.0%)	7/36 (19.4%)	0.08 (0.01–0.67)	
Sex				0.741
Male	1/75 (1.3%)	6/42 (14.3%)	0.12 (0.02–0.63)	
Female	1/77 (1.3%)	8/39 (20.5%)	0.09 (0.02–0.48)	
ASA classification				0.668
ASA I	1/82 (1.2%)	5/39 (12.8%)	0.13 (0.02–0.72)	
ASA II	1/70 (1.4%)	9/42 (21.4%)	0.09 (0.02–0.47)	
Operation duration				0.294
< 90 min	2/92 (2.2%)	5/38 (13.2%)	0.20 (0.05–0.78)	
≥90 min	0/60 (0.0%)	9/43 (20.9%)	0.07 (0.01–0.57)	
Preoperative MoCA				0.118
≥26	2/117 (1.7%)	4/45 (8.9%)	0.23 (0.07–0.80)	
< 26	0/35 (0.0%)	10/36 (27.8%)	0.06 (0.01–0.46)	

Adjusted ORs and 95% CIs were estimated using Firth penalized logistic regression because of the limited number of ePOCD events and the presence of zero-event cells in some strata. Models were adjusted for the prespecified covariates included in the primary multivariable model, excluding the variable defining the corresponding subgroup. *P* values for interaction were obtained from models including the corresponding GNRI × subgroup interaction term. Given the limited number of outcome events and multiple subgroup comparisons, all subgroup analyses were considered exploratory and hypothesis-generating. GNRI, Geriatric Nutritional Risk Index; ePOCD, early postoperative cognitive decline; OR, odds ratio; CI, confidence interval; ASA, American Society of Anesthesiologists; MoCA, Montreal Cognitive Assessment.

### Exploratory ROC Analysis of GNRI

The apparent AUC for GNRI in discriminating POD2 ePOCD was 0.731 (95% CI, 0.65–0.81). The cutoff maximizing the Youden index was 95.6, with corresponding sensitivity and specificity of 77.8% (95% CI 52.5–87.9) and 76.4% (95% CI 71.4–80.8), respectively. Bootstrap internal validation of the GNRI-only discrimination analysis using 1,000 resamples yielded an estimated optimism of 0.024, resulting in an optimism-corrected AUC of 0.707. Given the limited number of POD2 ePOCD events, these estimates should be considered exploratory and require external validation (Figure 2).

**Figure 2:**
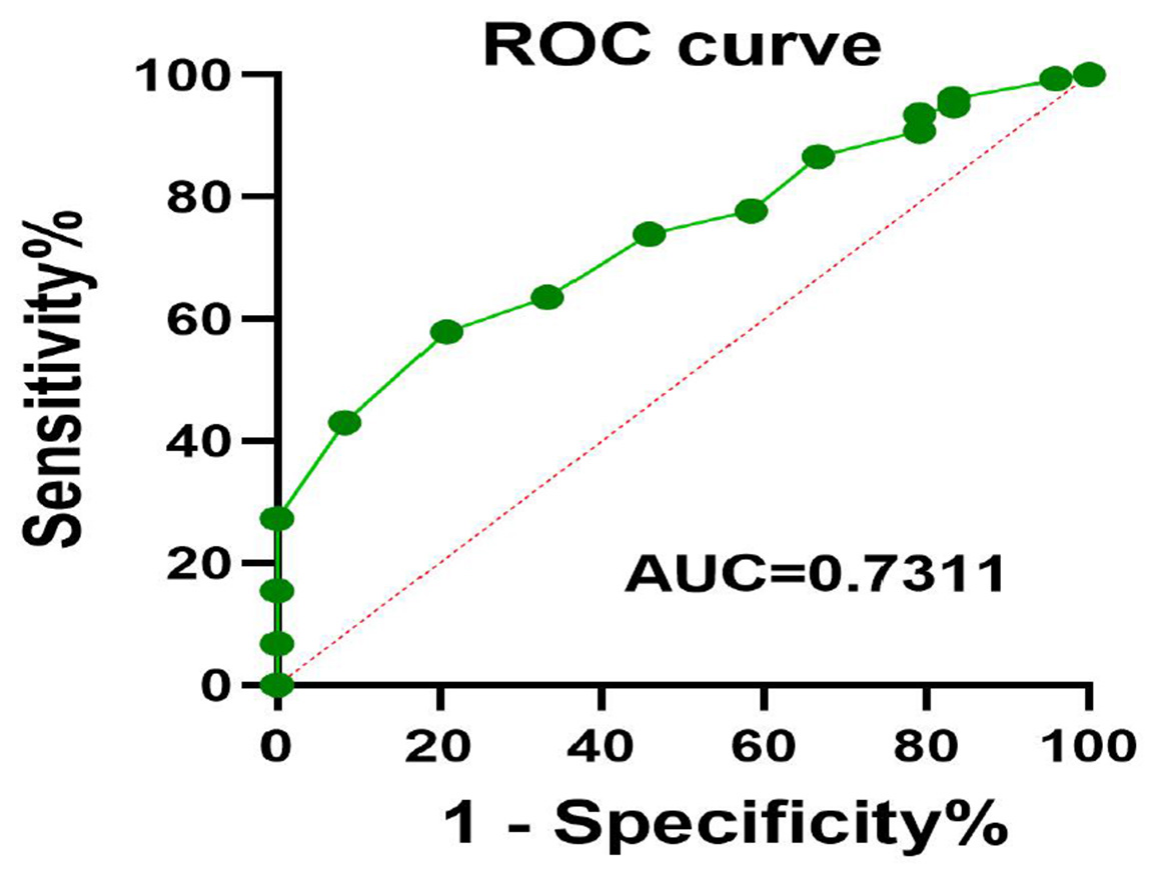
ROC curve of preoperative GNRI for discriminating POD2 ePOCD.

### Association between GNRI and POD2 IL-6

Consistently, Spearman's rank correlation analysis showed a negative correlation between preoperative GNRI and POD2 IL-6 levels (ρ = –0.12; 95% CI, −0.22 to −0.02; *P* = 0.022). These findings suggest an association between lower preoperative GNRI and higher POD2 IL-6 levels (Figure 3).

**Figure 3:**
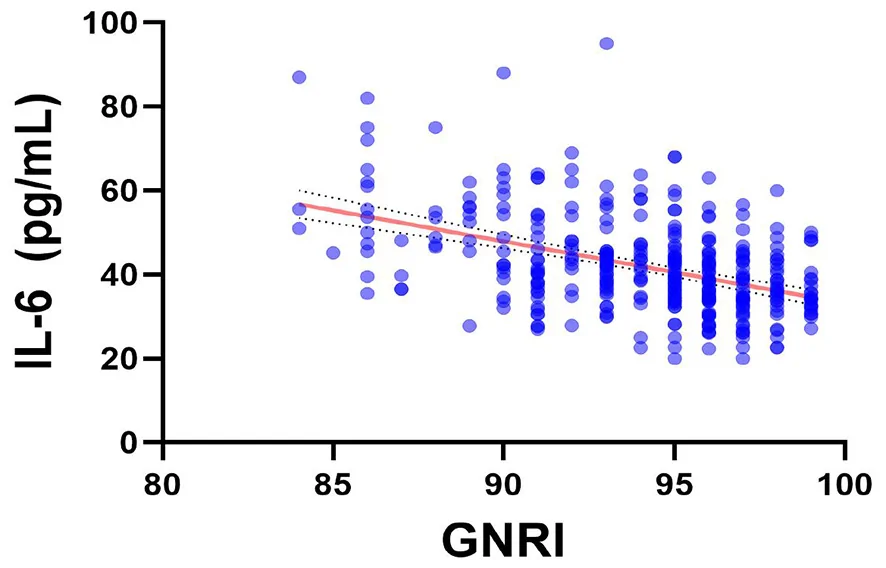
Correlation between preoperative GNRI and serum IL-6 level on postoperative day 2. Spearman rank correlation analysis was used to assess the association between GNRI and postoperative day 2 IL-6 levels (Spearman's ρ= −0.12, 95% CI [−0.22 to −0.02], *P* = 0.022).

## Discussion

This secondary analysis of a prospectively collected observational cohort included 361 elderly patients undergoing laparoscopic cholecystectomy and investigated the association between preoperative GNRI and early postoperative cognitive decline. Lower preoperative GNRI remained independently associated with higher odds of ePOCD after multivariable adjustment. These results suggest that preoperative nutritional status may represent an important factor related to early postoperative cognitive vulnerability in elderly patients.

An important consideration is the timing of postoperative cognitive assessment. In the present study, cognitive function was evaluated only on POD1 and POD2. Therefore, the observed cognitive changes should be interpreted as ePOCD rather than persistent postoperative neurocognitive dysfunction. Although early postoperative cognitive changes may be clinically relevant, they may also be influenced by transient factors such as residual anesthetic effects, postoperative pain, sleep disturbance, fatigue, acute physiological stress, and other perioperative factors. Accordingly, our findings should not be interpreted as evidence of persistent or long-term perioperative neurocognitive disorder. Longer-term follow-up using contemporary PND criteria will be necessary to determine whether early cognitive decline persists and whether it is associated with subsequent functional or cognitive outcomes.

The incidence of ePOCD in this study was 6.6%, and the incidence on postoperative day 1 was higher than that on postoperative day 2. This finding is consistent with previous studies reporting that cognitive changes are more frequently observed during the immediate postoperative period (7, 11, 19). Older adults exhibit age-related inherent physiological changes, including reduced organ function, decreased cognitive reserve, and impaired ability to maintain neurological homeostasis. Although laparoscopic cholecystectomy is a minimally invasive surgical procedure, it may still induce surgical stress responses stress responses, while anesthetic exposure may transiently influence central nervous system function, potentially contributing to early postoperative cognitive changes (20, 21). With recovery from surgical stress and gradual metabolism of anesthetic agents, cognitive performance may improve in some patients over time (22, 23). Therefore, early postoperative cognitive changes should be interpreted within the context of perioperative physiological stress rather than as equivalent to persistent neurocognitive disorders (24).

In the present study, patients with early postoperative cognitive decline exhibited lower preoperative GNRI values, and the incidence of cognitive decline increased across GNRI-defined nutritional risk categories. These findings indicate that poorer preoperative nutritional status is associated with increased susceptibility to early postoperative cognitive changes in elderly patients undergoing laparoscopic cholecystectomy. GNRI, calculated based on serum albumin concentration and body weight, provides a simple and practical approach for evaluating nutritional risk in older adults. Previous studies have demonstrated that GNRI is associated with postoperative complications, functional outcomes, and prognosis in elderly populations (25–28). Furthermore, low GNRI values have been linked to increased risks of postoperative neurological complications, including postoperative delirium, and other adverse clinical outcomes (29–32). Our findings extend these observations by suggesting a potential association between preoperative nutritional risk and early postoperative cognitive vulnerability.

Several biological mechanisms may contribute to the observed relationship between nutritional status and early postoperative cognitive decline. Malnutrition may impair physiological reserve, immune regulation, and recovery capacity, thereby increasing vulnerability to perioperative stress. Previous studies have suggested that nutritional impairment may influence drug metabolism, inflammatory responses, oxidative stress, and neuronal resilience, which may collectively affect postoperative cognitive recovery (33, 34). However, the present study was observational in nature, and the underlying mechanisms linking GNRI and cognitive changes cannot be conclusively determined. One possible explanation is that poor nutritional status may be associated with an enhanced postoperative inflammatory response, which has been implicated in postoperative cognitive decline. However, the relationship among GNRI, postoperative inflammation, and ePOCD requires further investigation.

Nutritional components and dietary interventions have also been suggested to influence cognitive recovery after surgery. Previous studies have reported that dietary supplementation with omega-3 fatty acids and micronutrients may improve neuroinflammation and synaptic function, thereby potentially reducing cognitive impairment after surgery (35–37). Malnutrition has been identified as a risk factor for postoperative delirium (38) and may contribute to adverse postoperative outcomes through impaired immune function, delayed recovery, increased systemic stress responses (39), and altered neurotransmitter metabolism (40, 41). Nevertheless, whether nutritional optimization before surgery can reduce the incidence of early postoperative cognitive decline requires confirmation through prospective interventional studies.

Univariate and multivariate logistic regression analyses revealed that lower preoperative GNRI and advanced age were associated with increased risk of ePOCD. Advanced age remains one of the most consistently reported risk factor for postoperative cognitive changes (42, 43). Aging is accompanied by structural and functional alterations in the brain, including reduced synaptic density, decreased cognitive reserve, and diminished tolerance to physiological stress, which may increase susceptibility to perioperative cognitive changes.

In addition, oxidative stress, inflammatory responses, and mitochondrial dysfunction have been proposed as potential contributors to nutrition-related cognitive impairment. These biological processes may influence synaptic function and neuroplasticity and contribute to neuronal vulnerability (34). In the present study, postoperative biomarker differences were explored as potential biological correlates of nutritional status; however, these findings should not be interpreted as evidence of a causal inflammatory pathway or mediation mechanism. Further studies incorporating longitudinal biomarker assessment and formal mechanistic analyses are needed to clarify the biological relationship between nutritional status, inflammation, and postoperative cognitive outcomes.

## Limitations

Several limitations should be acknowledged. First, this was a secondary analysis of a single-center prospectively collected observational cohort, which may limit the generalizability of the findings. Second, despite the overall sample size, the relatively small number of ePOCD events may have limited statistical power and increased the risk of overfitting in the multivariable analyses. Third, cognitive function was assessed only on postoperative days 1 and 2, precluding evaluation of longer-term cognitive outcomes. Fourth, the absence of an independent non-surgical retest control group prevented us from fully accounting for potential practice or retest effects. Fifth, residual or unmeasured confounding cannot be excluded despite multivariable adjustment. Sixth, the predictive performance of GNRI was evaluated within the same cohort without external validation and therefore requires confirmation in independent populations. Finally, the associations among GNRI, inflammatory biomarkers, and ePOCD were observational and should not be interpreted as evidence of causality. Future prospective, multicenter studies with larger numbers of ePOCD events, longer follow-up, appropriate control groups, and external validation are warranted.

## Conclusion

In conclusion, lower preoperative GNRI was associated with an increased risk of early postoperative cognitive decline in elderly patients undergoing laparoscopic cholecystectomy. These findings suggest that preoperative nutritional status may represent an important factor associated with postoperative cognitive vulnerability and may contribute to perioperative risk stratification in older surgical patients. However, given the observational nature of this study, the findings do not establish a causal relationship between nutritional status and cognitive outcomes, nor do they demonstrate that nutritional intervention can prevent postoperative cognitive decline. Further multicenter prospective studies and interventional trials are warranted to determine whether improving preoperative nutritional status can modify the risk of postoperative cognitive complications and to further elucidate the underlying mechanisms.

## Data Availability

The raw data supporting the conclusions of this article will be made available by the authors, without undue reservation.
